# Fetal Calf Serum Exerts an Inhibitory Effect on Replication of Duck Hepatitis A Virus Genotype 1 in Duck Embryo Fibroblast Cells

**DOI:** 10.3390/v12010080

**Published:** 2020-01-09

**Authors:** Minghang Wang, Lili Chai, Suyun Liang, Junfeng Lv, Lixin Yang, Shenghua Qu, Meiling Jin, Qingxiangzi Li, Xiaoyan Wang, Dabing Zhang

**Affiliations:** Key Laboratory of Animal Epidemiology of the Ministry of Agriculture, College of Veterinary Medicine, China Agricultural University, Beijing 100193, China; wangminghang@cau.edu.cn (M.W.); nth_tolos@163.com (L.C.); liangsu_yun@163.com (S.L.); junfeng1017@cau.edu.cn (J.L.); yanglixin@cau.edu.cn (L.Y.); qushenghua@cau.edu.cn (S.Q.); jml@cau.edu.cn (M.J.); s20183050722@cau.edu.cn (Q.L.)

**Keywords:** duck hepatitis A virus genotype 1, duck embryo fibroblast, cytocidal infection, plaque formation, fetal calf serum, inhibitory effect

## Abstract

Among the causative agents of duck viral hepatitis, duck hepatitis A virus genotype 1 (DHAV-1) is the most common virus reported in most outbreaks worldwide. How to propagate DHAV-1 in cell cultures efficiently remains a problem to be explored. Here, we aimed to test the effect of serum type on DHAV-1 replication in duck embryo fibroblast (DEF) cells. Comparative studies involved virus culture and passage, observation of cytopathic effect (CPE), virus quantification, and plaque formation assay. From the results of these investigations, we conclude that use of chicken serum (CS) in maintenance medium allows DHAV-1 to establish productive, cytocidal infection in DEF cells, whereas FCS exerts inhibitory effects on DHAV-1 replication, CPE development, and plaque formation. By using a neutralization test, we found that the direct action of FCS on virions is likely to play a key role in inhibiting DHAV-1 replication in DEF cells. Mechanism analyses revealed that FCS inhibits DHAV-1 replication at virus adsorption and reduces extracellular virus yields. The present work may shed light on a new perspective for antiviral agent development, and have provided a virus–host cell system for further studies on molecular mechanism involved DHAV-1 replication and pathogenesis.

## 1. Introduction

Duck hepatitis A virus genotype 1 (DHAV-1), historically known as duck hepatitis virus serotype 1 (DHV-1), was first isolated from an outbreak of duck viral hepatitis (DVH) in young White Pekin ducks on Long Island, New York, USA, in 1949. DHAV-1 usually affects ducklings under four weeks of age and may cause up to 95% mortality in ducklings less than one week old. The disease caused by DHAV-1 is characterized by lethargy, ataxia, sudden death with opisthotonos, and enlarged livers with punctate and ecchymotic hemorrhages [1]. DHAV-1 is currently classified within the species *Avihepatovirus A* in the genus *Avihepatovirus* of the family *Picornaviridae*, along with DHAV genotypes 2 and 3 [2,3,4]. There are other two viruses causing outbreaks of DVH, historically known as duck hepatitis virus serotypes 2 and 3 and currently classified within the genus *Avastrovirus* in the family *Astroviridae* [5,6,7,8,9]. Among the causative agents of DVH, DHAV-1 is the most common virus reported in most outbreaks worldwide [1,10,11].

The DHAV-1 genome consists of positive-sense, single-stranded RNA of approximately 7.7 kb. The polyadenylated genome contains a large open reading frame (ORF), encoding a polyprotein, which is preceded by a 5′ untranslated region (UTR) and followed by a 3′ UTR. The polyprotein was predicted to be cleaved into 10–12 mature products, forming its structural (VP0, VP3, and VP1) and nonstructural (2A, 2B, 2C, 3A, 3B, 3C, and 3D) proteins. A notable feature is the 2A protein, which contains three motifs/domains, including NPGP motif, AIG1-like conserved domain, and H-box/NC motif [12,13,14]. It is therefore suggested by Tseng et al. (2006) that DHAV-1 may possess three putative 2A proteins [14]. The 5′UTR was shown to possess a distinct hepacivirus/pestivirus-like internal ribosome entry site (IRES) [15].

Although there have been many reports describing the isolation and propagation of DHAV-1 in cell cultures of duck, chicken, and other avian embryo origins so far [14,16,17,18,19,20,21,22,23,24,25,26,27], how to propagate DHAV-1 in cell cultures efficiently remains a problem to be explored. It has been shown previously that the cytopathic effects (CPE) observed in some types of cell cultures were not of sufficient practical value for the development of an in vitro assay [23,28]. Of note, conflicting results were seen in previous publications as to whether DHAV-1 can be propagated and whether CPE can be produced in some types of cell cultures. For example, Hwang (1965) concluded that duck embryo fibroblast (DEF) cells might hold little promise for the development of a cytopathogenic strain of DHAV-1 or for propagation of the virus [29], whereas Golubnichi et al. (1976) reported successful growth and extensive CPE in DEF cells inoculated with chick embryo-adapted DHAV-1 [30]. Based on the description of Woolcock (1986), a CPE can only be produced in DEF cultures when attenuated virus is inoculated at a high titer [23].

Plaque assays for DHAV-1 have been developed by using primary duck embryo kidney (DEK) cells and duck embryo liver (DEL) cells [23,28,31]. In the study by Woolcock et al. (1982), the presence of fetal calf serum (FCS) in agarose overlay was shown to alter the diameter of the plaques formed in DEK cells [28]. Further study by Chalmers and Woolcock (1984) demonstrated that several mammalian (e.g., fetal calf, newborn calf, rabbit, and dog) sera have a nonspecific inhibitory effect on growth of DHAV-1 in DEK cells, whereas there is no or minimal inhibitory effect in sera from ducks or chickens [31]. Using an agarose overlay containing 2% chicken serum (CS) and 0.2% FCS, the study demonstrated that both virulent and attenuated DHAV-1 were shown to produce plaques in DEL cells [23]. It is unclear to date whether DHAV-1 can produce plaques in DEF cells.

In this study, we describe the investigation of growth properties of virulent and attenuated DHAV-1 strains in primary DEF cells, employing medium consisting of Dulbecco’s modified Eagle’s medium (DMEM) supplemented with 2% CS or FCS. Since FCS exerted an inhibitory effect on growth of DHAV-1 in DEF cells, we also investigated the mechanism of the FCS-mediated inhibition.

## 2. Materials and Methods

### 2.1. Cells, Viruses, and Antiserum

Primary DEF cells were prepared from 12-day-old embryonated Pekin duck eggs by a standard method [32] and maintained in growth medium consisting of DMEM (Macgene, Beijing, China) supplemented with 10% FCS (Corning, Corning, NY, USA), 100 U/mL of penicillin, and 0.1 mg/mL of streptomycin (10% FCS DMEM). The cells were grown in T25 flasks, seeded at 1 × 10^6^ cells per flask, or 24-well plates, seeded at 2 × 10^5^ cells per well, and incubated at 37 °C in a 5% CO_2_ atmosphere.

Two DHAV-1 strains were used: C-QYD, originally isolated in 10-day-old embryonated duck eggs from an outbreak of DVH and passaged once [33]; and C80, the 80th passage of the DHAV-1 C strain in specific pathogen-free (SPF) embryonated chicken eggs [12]. The viruses, present as embryo homogenates, were stored at −80 °C. Using embryonated duck and chicken eggs, we determined the titers of C-QYD and C80 to be 10^5.50^ and 10^6.84^ 50% embryo lethal dose (ELD_50_) per 0.1 mL, respectively.

Antiserum against DHAV-1 C-QYD was prepared in our laboratory previously and stored at −20 °C [34].

### 2.2. Virus Propagation

Confluent monolayers of DEF cells grown in T25 flasks were used to propagate C-QYD and C80. The virus-containing homogenates were clarified by centrifugation at 10,000× *g* for 10 min, followed by filtration through 0.22 μm Sterile Syringe Filters (Millipore, Billerica, MA, USA). The cells were washed three times with DMEM, and each of cultures was inoculated with 0.5 mL (10^4^ ELD_50_) of filtrate. After 1 h adsorption at 37 °C, the inoculum was removed, and maintenance medium consisting of DMEM supplemented with 2% CS (Solarbio, Beijing, China), 100 U/mL penicillin, and 0.1 mg/mL streptomycin (2% CS DMEM) was added. The inoculated cells were monitored daily, until 72 hpi or until an extensive CPE was observed. The cell culture (cells + supernatant) was subjected to three freeze–thaw cycles and clarified by centrifugation at 10,000× *g* for 10 min. The cell-free supernatants were harvested and passaged for an additional four times. Using 2% FCS DMEM as maintenance medium, both virulent and attenuated strains were passaged five times, as described above. The supernatant from each passage was tested by real-time quantitative PCR (RT-qPCR) assay following the protocol described below. In the following experiments (except the immunofluorescence test), the fifth passages of C-QYD and C80 cultured in 2% CS DMEM were used to inoculate DEF cells.

### 2.3. RT-qPCR Assay

The replication of DHAV-1 in DEF cells was determined by an RT-qPCR assay. For establishment of standard curve, reverse-transcription (RT)–PCR assay was performed to amplify a 214-bp cDNA fragment from the 5′UTR region of the C80 genome, employing previously reported reactions, conditions [35], and the qDHAV-1-f/r primer sets (Table S1) [36]. The PCR product was cloned into the pCloneEZ-Blunt-AMP/HC (Taihegene, Beijing, China) to construct recombinant plasmid pC-C80. Then, 2 μL from each of 10-fold dilutions (10^−3^–10^−7^; corresponding to 4.39 × 10^5^–43.9 copies/μL for pC-C80) of the recombinant plasmid was employed to generate standard curve with an AceQ qPCR SYBR Green Master Mix (Vazyme, Nanjing, China), according to the manufacturer’s instructions. Amplification was performed on an Applied Biosystems StepOne™ Real-Time PCR System (Thermo Scientific, Waltham, MA, USA), with the cycling conditions provided by the AceQ qPCR SYBR Green Master Mix. For virus quantification, RNA was extracted from 200 μL of each sample, using a TRIzol reagent (Thermo Scientific, Waltham, MA, USA) and dissolved in 50 μL of RNase-free water. Then, 5 μL of RNA was reverse transcribed into cDNA, using a HiScript First Strand cDNA Synthesis Kit and random hexamers (Vazyme, Nanjing, China). Then, 2 μL of cDNA was used for the determination of viral load. Each sample was detected in triplicate.

### 2.4. Determination of Viral Growth Kinetics

Confluent monolayers of DEF cells grown in 24-well plates were washed three times with DMEM and inoculated with virus at a multiplicity of infection (MOI) corresponding to 1 RNA copy/cell. Following 1 h of adsorption at 37 °C, the inoculum was removed, and 2% CS DMEM was added. Incubation at 37 °C in 5% CO_2_ atmosphere was continued. At 6, 12, 24, 36, 48, 60, and 72 h postinoculation (hpi), cells + supernatant and supernatant only were sampled respectively, and viral loads were detected by using the RT-qPCR assay, as described above.

### 2.5. Immunofluorescence Test

The fifth cell passages of C-QYD and C80 cultured in 2% CS DMEM and 2% FCS DMEM were subjected to the immunofluorescence assays. Confluent monolayers of DEF cells grown in 24-well plates were inoculated with virus, as described in Section 2.4, and 0.5 mL of 2% CS DMEM or 2% FCS DMEM was added. At 24 hpi, the cells were stained with chicken antiserum against DHAV-1 and fluorescein isothiocyanate (FITC)-conjugated goat anti-chicken IgY (KPL, Milford, MA, USA), as described previously [37]. The nuclei were stained with DAPI (Sigma-Aldrich, St Louis, MO, USA) for 5 min at room temperature. After being washed with phosphate-buffered saline three times, the cells were examined under fluorescence microscopy (Olympus, Tokyo, Japan), and the percentage of fluorescein-positive cells was determined by counting fluorescein- and DAPI-positive nuclei on digital images taken from three technical replicates.

### 2.6. Plaque Formation Assay

The plaque phenotype of DHAV-1 was determined, as described previously [37], employing an agarose overlay containing DMEM, 2% FCS or 2% CS, 1% low-melt agar (Macgene, Beijing, China), 100 U/mL of penicillin, and 0.1 mg/mL streptomycin (designated 2% FCS agarose overlay and 2% CS agarose overlay, respectively). The viruses were individually diluted with DMEM in 10-fold steps to 10^−8^. Confluent monolayers of DEF cells grown in 24-well plates were infected with virus as described in Section 2.4, except that 0.2 mL of diluted virus was used as inoculum. Following virus adsorption, the infected cells were divided into two groups: one group was overlaid with 0.5 mL of 2% CS agarose overlay, and the other with 0.5 mL of 2% FCS agarose overlay. After 60 h of incubation at 37 °C in a CO_2_ incubator, the cells were stained with crystal violet for observation of plaque morphology, as described previously [38].

### 2.7. Neutralization Test

The effect of FCS on DHAV-1 was investigated following the protocol used in neutralization test [37], employing CS as a control. Briefly, three dilutions of serum were individually mixed with an equal volume of virus (2 × 10^5^ RNA copies), to produce three serum concentrations (0.5%, 1%, and 2%). After incubation at 37 °C for 1 h, the serum–virus mixtures were used as inocula to inoculate DEF cells grown in 24-well plates. Following 1 h of adsorption at 37 °C, the inocula were removed, and the cells were washed three times with DMEM. Next, 0.5 mL of maintenance medium was added to each well. The cell cultures were harvested at 24 hpi for C80 and at 36 hpi for C-QYD and tested for viral loads by using the RT-qPCR assay. Apart from FCS described above, which was sourced from South America, another two FCS products, which were sourced from Australia (Corning, Corning, NY, USA) and North America (Macgene, Beijing, China), were also used for analysis.

### 2.8. Assays for Effect of Pre-Incubation of Cells with FCS on Virus Infection

Confluent monolayers of DEF cells grown in 24-well plate were washed three times with DMEM. Next, 0.2 mL of 2% FCS DMEM was added onto the cultures, and 2% CS DMEM was included as a control. After incubation at 37 °C for 1 h, DEF cells were infected with DHAV-1, as described in Section 2.4. After the cells were washed again, 0.5 mL of 2% CS DMEM was added to each well. Incubation at 37 °C in a CO_2_ incubator was continued. At 18 hpi, cell cultures were sampled for detection of viral loads.

### 2.9. Virus Attachment Inhibition Assay

The serum–virus mixture (0.4 mL) was prepared by addition of FCS or CS and virus (6 × 10^5^ copies) in pre-cold DMEM, to give a final serum concentration of 2%. DEF cells were grown in 12-well plates (6 × 10^5^ cells/well). When confluent, the cultures were placed at 4 °C for 30 min and washed three times with pre-cold DMEM. Subsequently, the cells were inoculated with the serum–virus mixture. Following 1 h of adsorption, unbound viruses were removed by washing three times with pre-cold DMEM. The cells were dissolved in 1 mL of TRIzol reagent and sampled for detection of viral loads.

### 2.10. Virus Internalization Inhibition Assay

Confluent monolayers of DEF cells grown in 12-well plates (6 × 10^5^ cells/well) were washed three times with pre-cold DMEM and inoculated with DHAV-1 at a MOI of 1 copy/cell. After incubation at 4 °C for 1 h, unbound viruses were removed by washing three times with pre-cold DMEM, and 0.4 mL of maintenance medium was added to each culture. Following incubation at 37 °C for 1 h, the cells were washed again and treated with 0.4 mL of proteinase K (0.25 mg/mL) (Solarbio, Beijing, China) at 4 °C for 30 min, to remove extracellular viruses. Subsequently, the activity of proteinase K was inactivated by the addition of phenyl methyl sulfonyl fluoride (PMSF) (Solarbio, Beijing, China) at a final concentration of 2 mM/L. The cell cultures were harvested and subjected to centrifugation at 2000× *g* for 10 min. After washing three times with DMEM, the cell pellets were resuspended in 1 mL of TRIzol reagent, for detection of viral loads.

### 2.11. Assays for Effect of FCS on Viral RNA Replication

The assays were performed to investigate the effect of FCS on viral RNA replication, employing CS as a control. Confluent monolayers of DEF cells grown in 24-well plates were infected with virus as described in Section 2.4. Following 1 h of adsorption at 37 °C, 0.5 mL of maintenance medium was added to each well. After incubation at 37 °C for 5 h, the medium was removed, and the cells were dissolved in 1 mL of TRIzol reagent for RNA extraction. RNA was reverse transcribed using reverse primer qDHAV-1-f (Supplementary Table S1). The amount of negative strand RNA was quantified with RT-qPCR.

### 2.12. Assays for Effect of FCS on DHAV-1 IRES-Driven Translation

The assays were performed as follows. The full length 5′UTR was amplified from C-QYD by RT-PCR, using primers DHAV-1-5′UTR-f and C-QYD-5′UTR-r (Table S1). Using the PCR product as a template, we introduced the homology arm to the C-QYD 5′UTR by PCR with rDHAV-1-5′UTR-f and rC-QYD-5′UTR-r (Table S1). The SalI and SacI restriction enzymes were used to digest the p2Luc vector (BioVector NTCC Inc, Beijing, China), which contained reporter genes *renilla* luciferase (Rluc) and firefly luciferase (Fluc). The PCR product was then cloned into p2Luc by using a homologous recombination kit (Vazyme, Nanjing, China). The resulting recombinant plasmid was designated pRF-C-QYD. Similarly, the recombinant plasmid pRF-C80, which contained the C80 5′UTR and the homology arm, was constructed. The recombinant plasmids were designed to allow initiation of the Fluc synthesis to be directed by DHAV-1 IRES, whereas the translation initiation of Rluc was dependent on cap structure.

Confluent monolayers of DEF cells grown in 24-well plates were washed three times with DMEM. The recombinant plasmid was transfected into the cells (500 ng/well), using a Lipofectamine 3000 Transfection Reagent (Invitrogen, Carlsbad, CA, USA). The cells were incubated at 37 °C in a CO_2_ incubator. Eighteen hours later, the supernatant was removed, and the cells were washed three times with DMEM. The cells received maintenance medium (0.5 mL/well) and were incubated at 37 °C. Six hours later, the supernatant was removed, and the cells were washed again. The cells were lysed with Passive Lysis Buffer. Then, 20 μL of lysis product was tested for the activity of Rluc and Fluc by a Dual-Luciferase Reporter Assay System (Promega, Madison, WI, USA), using a GloMax 20/20 Luminometer (Promega, Madison, WI, USA). The DHAV-1 IRES activity was expressed as percentage of Fluc activity normalized by Rluc activity.

The recombinant plasmid was linearized with KpnI restriction enzyme and calf intestinal alkaline phosphatase (NEB, Ipswich, MA, USA). The linearized plasmid DNA was purified by using a DNA Fragment Purification Kit (Takara Bio, Kyoto, Japan) and transcribed into capped RNA in vitro, using an mMESSAGE mMACHINE T7 Transcription Kit (Ambion, Austin, TX, USA). After monolayers of DEF cells were maintained in 24-well plates and washed three times with DMEM, the cells received 0.5 mL of maintenance medium. Next, the capped RNA was transfected into the cells (500 ng/well), using a Lipofectamine MessengerMAX mRNA Transfection Reagent (Invitrogen, Carlsbad, CA, USA). The cells were incubated at 37 °C in a CO_2_ incubator. Seventeen hours later, the activity of Rluc and Fluc was measured, as described above.

In the abovementioned transfection experiments, the effect of FCS on DHAV-1 IRES-driven initiation of Fluc synthesis was assessed to reflect the effect of FCS on translation initiation of DHAV-1 protein. CS served as a control.

### 2.13. Assays for Effect of FCS on Extracellular Virus Yields

Confluent monolayers of DEF cells grown on 24-well plates were infected with virus, as described in Section 2.4. Next, the cultures were divided into four groups: the first and second groups received 2% FCS DMEM and 2% CS DMEM, respectively, and the third and fourth groups received 0.5 mL of DMEM. Following incubation at 37 °C for 1 h, the third and fourth groups received FCS and CS (final concentration of 2%), respectively. Incubation at 37 °C in a CO_2_ incubator was continued. The supernatant was sampled at 24 hpi for C80 and at 36 hpi for C-QYD and tested for viral loads.

### 2.14. Statistical Analysis

For each time point and treatment, samples were collected from three biological replicates. Data were calculated as means ± standard deviation (SD). Differences between groups were analyzed by using an independent samples t-test implemented in the SPSS Statistics 21th software (IBM, Armonk, NY, USA). A *p* < 0.05 value was considered statistically significant.

## 3. Results

### 3.1. Replication Efficiency of DHAV-1 in DEF Cells Maintained in Medium Containing Chicken Serum (CS)

**Figure 1:**
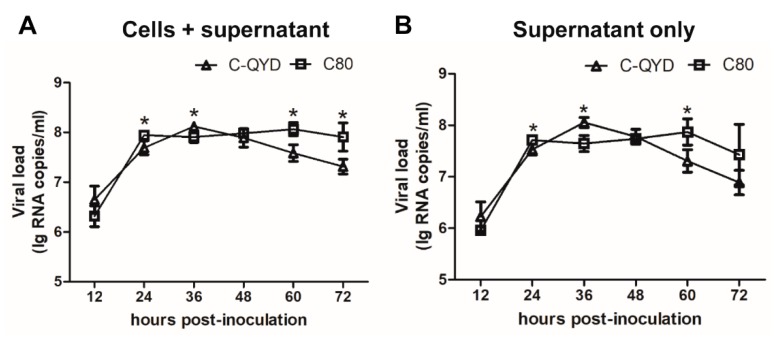
Growth kinetics of DHAV-1 in DEF cells. The fifth cell passage viruses of C-QYD and C80 were used to inoculate DEF cells at a MOI corresponding to one copy/cell. Viral yields in DEF cells were reflected by RNA copies detected with RT-qPCR. At each time point, cells + supernatant (**A**) and supernatant only (**B**) were respectively collected from three wells of 24-well plates. Error bars represent the SD (*n* = 3); * *p* < 0.05.

It has been shown previously that fetal calf serum (FCS) has a nonspecific inhibitory effect on DHAV-1, whereas there is no or minimal inhibitory effect in CS [31]. Thus, we attempted to propagate DHAV-1 by using 2% CS DMEM. To investigate the replication efficiency of DHAV-1 in DEF cells, the growth curves were established by using virulent C-QYD and attenuated C80 strains. In cell cultures (cells + supernatant) and supernatants, the titers of C-QYD and C80 peaked at 36 (10^8.12±0.07^ and 10^8.05±0.10^ RNA copies/mL) and 24 hpi (10^7.95±0.01^ and 10^7.71±0.02^ RNA copies/mL), respectively. Since then, the viral titers showed the tendency to decline. Relatively high levels of viral load were detectable at 72 hpi in both cell cultures (C-QYD: 10^7.31±0.15^ RNA copies/mL; C80: 10^7.91±0.28^ RNA copies/mL) and supernatants (C-QYD: 10^6.89±0.24^ RNA copies/mL; C80: 10^7.43±0.59^ RNA copies/mL) (Figure 1A,B). These results indicate that both virulent and attenuated DHAV-1 strains replicate efficiently in DEF cells using maintenance medium containing 2% CS.

### 3.2. Effect of FCS on DHAV-1 Infection in DEF Cells in Comparison with CS

**Figure 2:**
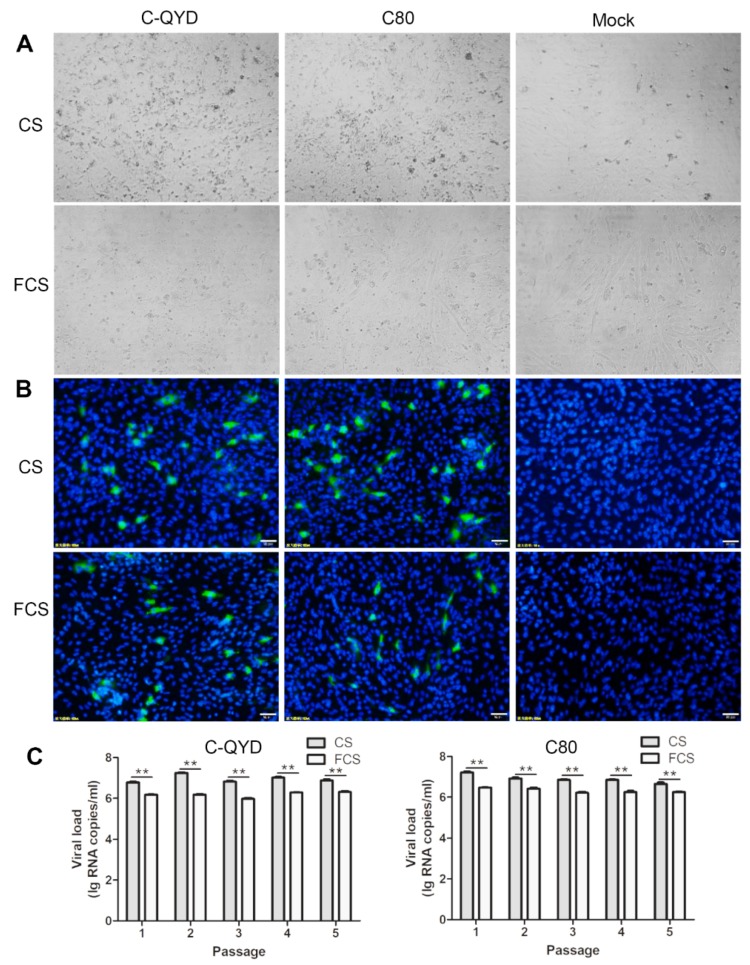
Characterization of DHAV-1 growth in DEF cells. (**A**) DEF cells after inoculation with DHAV-1. Upper panel, the third cell passage of C-QYD and the second cell passage of C80 were used as inocula. Two percent CS DMEM served as maintenance medium. The cells were checked at 24 hpi. Lower panel, the fifth passages of C-QYD and C80 were used as inocula. Two percent FCS DMEM served as maintenance medium. The cells were checked at 72 hpi. (**B**) Immunofluorescence showing in DEF cells 24 h after inoculation with the fifth cell passage viruses of C-QYD and C80. Upper panel, 2% CS DMEM served as maintenance medium; lower panel, 2% FCS DMEM served as maintenance medium. The analysis was conducted by using specific chicken antisera against C-QYD and a FITC-conjugated goat anti-chicken IgY, followed by nuclear staining with DAPI. Bar = 50 μm. (**C**) Titers of the first to fifth cell passage viruses of C-QYD and C80. DEF cell cultures 24–72 h after inoculation with virus were sampled and tested by using RT-qPCR assay. Error bars represent the SD (*n* = 3); ** *p* < 0.01.

To investigate the effect of FCS on DHAV-1 infection in DEF cells in comparison with CS, we assessed CPE and viral load during virus passages and performed indirect immunofluorescence (IIF) test with the fifth passage viruses. With 2% CS DMEM, both C-QYD and C80 strains produced a noticeable CPE in DEF cells after two to three passages (Figure 2A, upper panel). Following further passages, more pronounced CPE, including cell shrinkage and detachment from the surface of the flask, was observed between 24 and 36 hpi. When 2% FCS was used in maintenance medium, however, neither virulent nor attenuated strains induced noticeable CPE following five passages (Figure 2A, lower panel). In RT-qPCR and IIF assays, DHAV-1 was shown to grow in DEF cells maintained in both media (Figure 2B,C). However, viral loads of the first to fifth cell passage viruses generated from 2% FCS DMEM (C-QYD: 10^5.98±0.07^–10^6.31±0.08^ RNA copies/mL; C80: 10^6.22±0.08^–10^6.47±0.03^ RNA copies/mL) were significantly lower than those from 2% CS DMEM (C-QYD: 10^6.65±0.03^–10^7.22±0.08^ RNA copies/mL; C80: 10^6.78±0.08^–10^7.25±0.06^ RNA copies/mL) (Figure 2C). Moreover, the fluorescein-positive rates produced from 2% FCS DMEM (C-QYD: 3.182 ± 0.162%; C80: 2.704 ± 1.034%) were significantly lower than those from 2% CS DMEM (C-QYD: 4.742 ± 0.809%; C80: 4.831 ± 0.410%) (Figure 2B and Figure S1). Together, these data indicate that FCS exerts an inhibitory effect on the replication and CPE development of DHAV-1 in DEF cells.

### 3.3. Effect of Serum Type on Formation of DHAV-1 Plaques in DEF Cells

**Figure 3:**
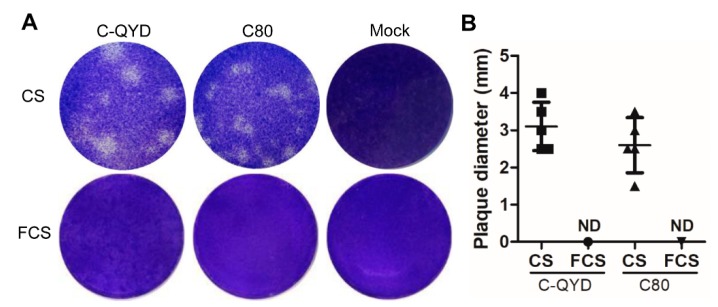
Effect of serum type on formation of plaques in DHAV-1-infected DEF cells. (**A**) DHAV-1 plaques in DEF cells overlaid with agarose overlays containing CS and FCS, respectively. Plaques were visualized by crystal violet staining at 60 hpi. (**B**) Comparison of the effect of CS and FCS on diameters of DHAV-1 plaques in DEF cells. ND: Not detectable.

To test whether serum type has an impact on plaque formation in DHAV-1-infected DEF cells, we conducted plaque assays by using agarose overlays containing CS or FCS. DHAV-1 produced clear plaques when 2% CS was used (Figure 3A, upper panel). There was no significant difference in diameter between C-QYD (3.1 ± 0.65 mm) and C80 (2.5 ± 0.79 mm) (Figure 3B). Even though the viruses were diluted 10^5^-fold, the plaques were still visible. However, no plaques were produced in DEF cells by both C-QYD and C80 viruses at a high concentration (1:100 dilution) when CS was replaced by FCS in the agarose overly (Figure 3A, lower panel). The data indicate that FCS has an inhibitory effect on the formation of DHAV-1 plaques in DEF cells.

### 3.4. Inhibitory Effect of FCS on DHAV-1

**Figure 4:**
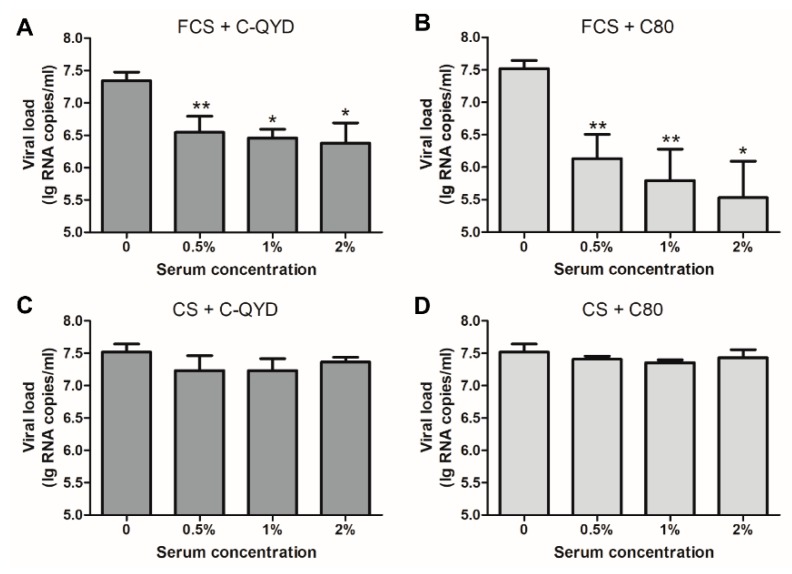
Quantitation of DHAV-1 genome replication in DEF cells inoculated with serum–virus mixture. The DHAV-1 C-QYD and C80 strains were individually preincubated with three concentrations (0.5%, 1%, and 2%) of FCS (**A**,**B**) and CS (**C**,**D**) at 37 °C for 1 h. DMEM–virus mixture was included as control. For each serum concentration, samples (cells + supernatant) were collected from three wells of 24-well plates. Viral load in each sample was quantified by RT-qPCR. Error bars represent the SD (*n* = 3); * *p* < 0.05; ** *p* < 0.01.

To investigate whether FCS has an effect on DHAV-1, infection experiments were conducted by using serum–virus mixtures as inocula, as performed in the neutralization assay. Compared to untreated controls, preincubation of DHAV-1 with FCS at final concentrations of 0.5%, 1%, and 2% resulted in decreases in viral load by 86.8%, 89.3%, and 90.5% for C-QYD (Figure 4A), and by 95.9%, 98.1%, and 99.0% for C80 (Figure 4B), respectively. By contrast, preincubation with CS showed no significant effect on viral load of both C-QYD and C80 when compared with untreated controls (*p* > 0.05) (Figure 4C,D). As expected, FCS sourced from Australia and North America exhibited an inhibitory effect on DHAV-1 replication in a dose-dependent manner (Figure S2). Conclusively, FCS may contain a virus-inhibitory substance(s) which exerts a significant inhibitory effect on DHAV-1 replication in DEF cells in a dose-dependent manner.

### 3.5. Effect of FCS on Virus Adsorption and Penetration

**Figure 5:**
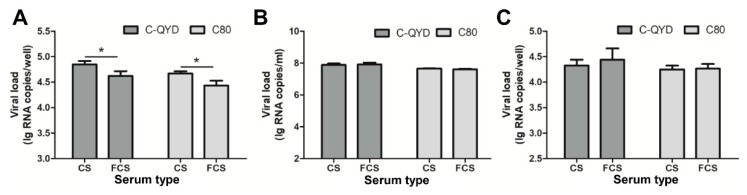
Investigation into the effect of FCS on virus adsorption and penetration. (**A**) Virus attachment inhibition assay. The FCS–virus mixture was used as inoculum. After adsorption at 4 °C for 1 h, the cells were washed to remove unbound virus, and the amount of bound virus was quantified. (**B**) DEF cells were pretreated with 2% FCS DMEM at 37 °C for 1 h, followed by inoculation with virus. Viral loads in cells + supernatant were detected at 18 hpi. (**C**) Analysis of effect of FCS on virus entry. Virus adsorption was carried out at 4 °C, followed by incubation of the cells with FCS at 37 °C for 1 h. Proteinase K was employed to remove extracellular viruses and inactivated with PMSF. Intracellular viruses were quantified. In each test, CS served as a control. Error bars represent the SD (*n* = 3); * *p* < 0.05.

To test whether FCS has an impact on adsorption of DHAV-1 to cell surface, we conducted a viral attachment inhibition assay. In this analysis, virus–serum mixtures were used as inocula, and the inoculated DEF cells were incubated at 4 °C for 1 h, to allow adsorption of virus to cell surface. As shown in Figure 5A, viral loads detected in the FCS-treated groups were significantly lower than those in the CS-treated groups (*p* < 0.05), suggesting that FCS exerts an inhibitory effect on DHAV-1 replication at virus adsorption.

To investigate whether FCS blocks virus adsorption by binding of component(s) in the serum to the cell surface or by affecting receptors that DHAV-1 binds to, we conducted infection experiments after preincubation of DEF cells with FCS at 37 °C for 1 h. High levels of viral load were detectable in FCS-treated cultures at 18 hpi for both C-QYD and C80, which were comparable to those obtained from the CS-treated groups (*p* > 0.05; Figure 5B). The data suggest that FCS has no blocking effect on adsorption of DHAV-1 to the cell surface by binding to or affecting the receptor sites.

To investigate whether FCS inhibits penetration of DHAV-1 into the interior of DEF cells, we carried out virus adsorption at 4 °C, followed by incubation of the cells with FCS at 37 °C. As shown in Figure 5C, viral loads of C-QYD and C80 in FCS-treated groups were comparable to those derived from control group (*p* > 0.05). The data indicate that FCS has no or little effect on entry of DHAV-1 into DEF cells.

### 3.6. Effect of FCS on Gene Expression of DHAV-1

**Figure 6:**
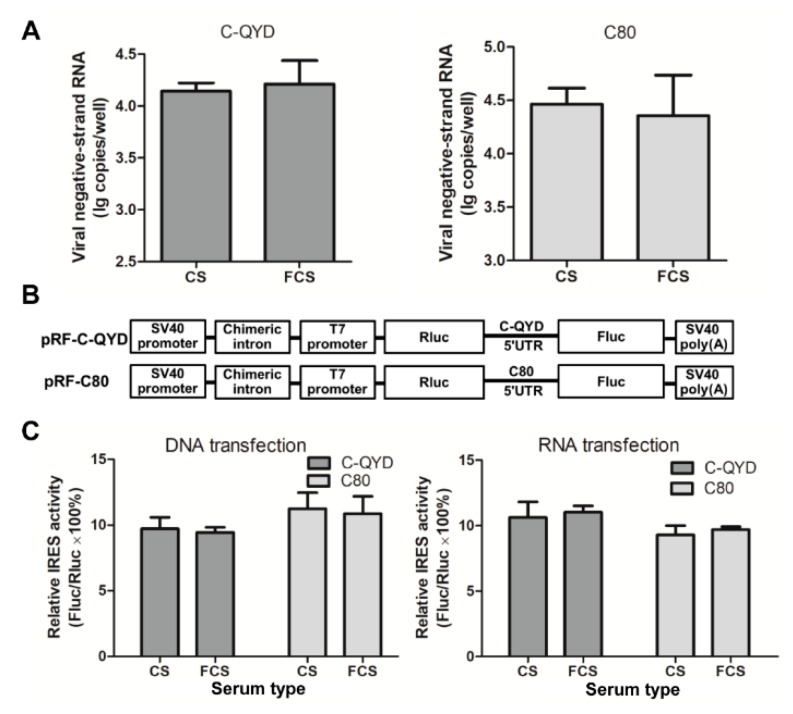
Analysis of effect of FCS on gene expression in DHAV-1-infected DEF cells. (**A**) Determination of effect of FCS on RNA replication. The negative strand replicative–intermediate of DHAV-1 was quantified at 6 hpi. (**B**) Schematic diagram of bicistronic reporter plasmids. (**C**) Analysis of effect of FCS on DHAV-1 IRES-mediated translation. Left panel, DEF cells were transfected with the bicistronic DNA plasmids. The activity of Fluc and Rluc was measured at 24 h after transfection. Right panel, DEF cells were transfected with in vitro transcribed RNA. The activity of Fluc and Rluc was measured at 17 h after transfection. Error bars represent the SD (*n* = 3).

To test whether FCS inhibits viral RNA replication, the negative strand replicative–intermediate of DHAV-1 was quantified at 6 hpi. Incorporation of FCS and CS in maintenance medium produced comparable amounts of negative strand RNA in DEF cells infected by C-QYD and C80 (*p* > 0.05; Figure 6A), indicating that FCS may have no or little effect on intracellular RNA replication.

To investigate whether FCS inhibits initiation of viral protein directed by DHAV-1 IRES, recombinant plasmids encoding for the Rluc and Fluc reporters were produced. The initiation of Rluc synthesis is dependent on 5′ terminal cap structure, and the initiation of Fluc synthesis is directed by DHAV-1 IRES (Figure 6B). At 24 h after transfection of DEF cells with both pRF-C-QYD and pRF-C80, comparable relative IRES activities (Fluc/Rluc × 100%) were found in DEF cells maintained in 2% FCS DMEM and 2% CS DMEM (*p* > 0.05; Figure 6C, left panel). When capped RNAs transcribed in vitro from plasmid DNAs were employed to transfect DEF cells, similar results were also obtained (*p* > 0.05; Figure 6C, right panel). Taken together, these data indicate that FCS has no inhibitory effect on internal initiation of protein synthesis directed by DHAV-1 IRES.

### 3.7. FCS-Mediated Inhibitory Effect on Extracellular Virus Yields

**Figure 7:**
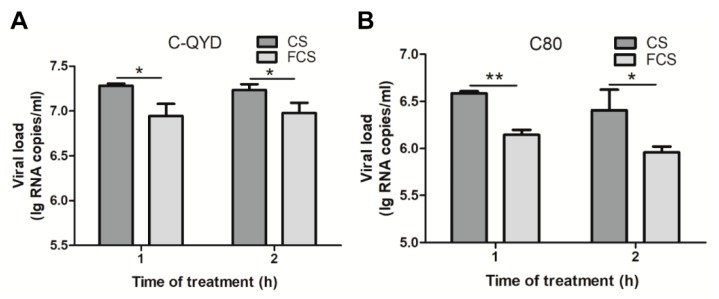
Analysis of effect of FCS on extracellular viral loads. DEF cells inoculated with C-QYD (**A**) or C80 (**B**) were maintained with DMEM and treated with FCS at 37 °C by addition of FCS into the wells at 1 and 2 hpi respectively, and CS was included as a control. The supernatants were collected from three well of 24-well plates, and the viral loads were quantified by RT-qPCR assay. Error bars represent the SD (*n* = 3); * *p* < 0.05; ** *p* < 0.01.

To test whether FCS has an effect on extracellular virus yields, we detected viral loads in supernatants of infected DEF cells maintained in maintenance medium at 1 and 2 hpi, respectively. In this analysis, viral loads were detected at 24 hpi for C80 and 36 hpi for C-QYD, which were determined on the basis of replication kinetics (Figure 1B). Compared with CS, addition of FCS in maintenance medium at both one and two hours after inoculation resulted in significant decreases in viral loads in supernatants (Figure 7A,B) (*p* < 0.05). These data indicate that FCS has significant inhibitory effect on extracellular virus yields.

## 4. Discussion

In this study, we described the effect of sera type on replication of DHAV-1 in primary DEF cells. Based on comparisons of CPE development, viral loads detected from the first to fifth cell passage viruses, and fluorescein-positive rates obtained from the infected cells, it is concluded that use of CS in maintenance medium allows DHAV-1 to establish productive, cytocidal infection in DEF cells, whereas FCS exerts inhibitory effects on DHAV-1 replication and CPE development.

It has been documented that FCS in agarose overlay affects sizes of DHAV-1 plaques formed in DEL and DEK cells in comparison with sera from chickens and ducks [23,28,31]. We showed that FCS dramatically inhibited plaque formation of DHAV-1 in DEF cells, whereas clear plaques formed in the cells when CS was used in the agarose overlay. These findings support the view that FCS may be related to the change of cytocidal infection to noncytocidal infection in DEF cells, and FCS is likely to exert the inhibitory effect on DHAV-1 plaque formation in a cell-type-independent manner. The fact that FCS from three different geographic areas displayed the inhibitory effect on DHAV-1 suggests that the virus-inhibitory substance may exist widely in fetal calf sera produced in different regions. The inhibitory effect of FCS on virus replication and plaque formation may help to explain previous unsuccessful propagation of DHAV-1 in cell cultures.

In the investigation of mechanisms of FCS-mediated inhibitory effect on DHAV-1 replication, preincubation of the virus with FCS dramatically reduced the viral yields. This suggests that the direct action of FCS on viral particles may play a key role in inhibiting DHAV-1 replication in DEF cells. The present observations are in agreement with previous findings of Chalmers and Woolcock (1984), who reported that the inhibitory effect of FCS on DHAV-1 replication might be attributed to a dramatic neutralizing effect on virus [31]. Viral attachment inhibition assay suggested that FCS inhibits DHAV-1 replication at virus adsorption. Based on the data obtained from infection experiment after preincubation of DEF cells with FCS, we conclude that FCS has no blocking effect on virus adsorption by binding to or affecting the receptor sites.

In addition to adsorption of DHAV-1 to the cell surface, this work has also shown that FCS exerts an inhibitory effect on extracellular virus yields. It is likely that the direct action of FCS on viral particles may reduce the amounts of progeny viruses to infect other cells. At present, it is uncertain whether the effect of FCS on virus loads in supernatant is associated with FCS-mediated inhibitory impact on virus assembly and release. A recent work in our laboratory showed that FCS exerts an inhibitory effect on DHAV-3 through the same mechanisms [37], suggesting that an FCS-mediated inhibitory effect on DHAV replication in cell cultures shows no genotype specificity. Collectively, these data provide a promising strategy for the development of a potent inhibitor against DHAV by further biochemical analysis of FCS.

Earlier work in our laboratory showed that the use of CS in maintenance medium is of help to the replication of DHAV-3 in DEF cells. Unlike DHAV-1, DHAV-3 cannot produce cytopathic changes in DEF cells [37]. Thus, the present observations further demonstrated that DHAV-1 and DHAV-3 infections lead to disparate effects on DEF cells. Although both DHAV-1 and DHAV-3 cause outbreaks of DVH with similar high mortality, clinical signs, and gross lesions, their genome sequences differ greatly from each other (genome: 70–73%; polyprotein: 77–86%). Furthermore, the 5′ and 3′ UTRs and VP1 of the two viruses differ in length [3,12,13,14,33,39]. Therefore, it is likely that the disparate infection types established by the two DHAV genotypes might be attributed to differences in their genomic sequence, which remains to be clarified in the future.

Taken together, we have shown that FCS exerts an inhibitory effect on replication of DHAV-1 in DEF cells. The analysis on the inhibitory mechanism revealed that FCS can reduce the amounts of virus binding to cell surface and extracellular virus yields. The present work may shed light on a new perspective for antiviral-agent development and have provided a virus–host cell system for further studies on molecular mechanism involved DHAV-1 replication and pathogenesis.

## Supplementary Materials

The following are available online at https://www.mdpi.com/1999-4915/12/1/80/s1. Figure S1: Immunofluorescent staining of DHAV-1-infected DEF cells at 24 h post-inoculation. Figure S2: Analysis of effect of FCS sourced from Australia and North America on DHAV-1 replication. Table S1: Primers used in this study.
