# Supplementary material for: Fetal Calf Serum Exerts an Inhibitory Effect on Replication of Duck Hepatitis A Virus Genotype 1 in Duck Embryo Fibroblast Cells
Source: Viruses. 2020 Jan 9;12(1):80. doi: 10.3390/v12010080 (PMC7019637; doi:10.3390/v12010080)
Supplement: Supplementary file 1 [file viruses-12-00080-s001.zip › Supplementary Files/Supplementary Figures.pdf]

Supplementary Figure 1

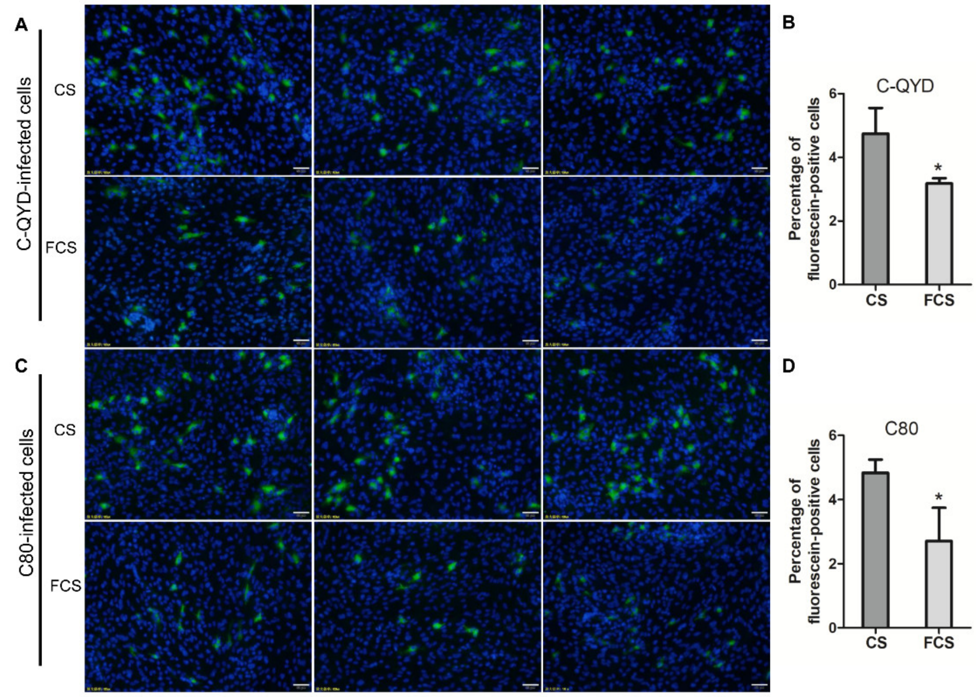

Figure S1. Immunofluorescent staining of DHAV-1-infected DEF cells at 24 h post-inoculation. The fifth cell passages of C-QYD (**A**) and C80 (**C**) cultured in 2% CS DMEM and 2% FCS DMEM were employed. Upper panel, 2% CS DMEM served as maintenance medium; lower panel, 2% FCS DMEM served as maintenance medium. The images were taken from three technical replicates. The percentage of fluorescein-positive cells derived from C-QYD (**B**) and C80 (**D**) infected groups was determined by counting fluorescein- and DAPI-positive nuclei. Bar = 50 μm. Error bars represent the SD (n=3). *, *P*<0.05.

Supplementary Figure 2

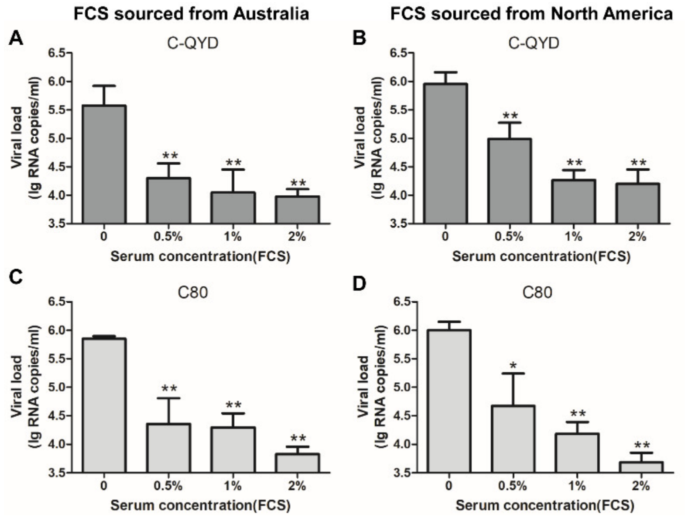

Figure S2. Analysis of effect of FCS sourced from Australia and North America on DHAV-1 replication. C-QYD and C80 strains were individually preincubated with three concentrations (0.5, 1, and 2%) of FCS sourced from Australia (**A** and **C**) and North America (**B** and **D**). DMEM-virus mixture was

included as control. Samples (cells + supernatant) were collected at 36 hpi for C-QYD and 24 hpi for C80, and viral load in each sample was quantified by RT-qPCR. Error bars represent the SD (n=3). *, $P<0.05$. **, $P<0.01$.
