# Supplementary material for: Fetal Calf Serum Exerts an Inhibitory Effect on Replication of Duck Hepatitis A Virus Genotype 1 in Duck Embryo Fibroblast Cells
Source: Viruses. 2020 Jan 9;12(1):80. doi: 10.3390/v12010080 (PMC7019637; doi:10.3390/v12010080)
Supplement: Supplementary file 1 [file viruses-12-00080-s001.zip › Supplementary Files/Supplementary Table.pdf]

Supplementary Table 1

Table S1. Primers used in this study

Primer[a]	Sequence (5′→3′)[b]	Product length (bp)
qDHAV-1-f	GGAGGTGGTGCTGAAATAT	214
qDHAV-1-r	CATGTGCCTGGACAGAT	
DHAV-1-5'UTR-f	GTAATTGAAAGCGGGTGCATG	632
C-QYD-5'UTR-r	CATTGCATGAAAGCCTACTGGTAT	
C80-5'UTR-r	CATTTCATGAAAGTCTACTGGTATTATAGTG	
rDHAV-1-5'UTR-f	caaaaatgaacaaatgtcgacGTAATTGAAAGCGGGTGCATG	674
rC-QYD-5'UTR-r	gtttttggcgtcttcgagctcCATTGCATGAAAGCCTACTGGTAT	
rC80-5'UTR-r	gtttttggcgtcttcgagctcCATTTCATGAAAGTCTACTGGTATTATAGTG	

[a] f: forward primer; r: reverse primer.

[b] lowercase letters represent homology arms necessary for homologous recombination.
